# Genetic diversity of *Mycobacterium tuberculosis* isolates from Tochigi prefecture, a local region of Japan

**DOI:** 10.1186/s12879-017-2457-y

**Published:** 2017-05-25

**Authors:** Fuminori Mizukoshi, Tohru Miyoshi-Akiyama, Hiroki Iwai, Takako Suzuki, Reiko Kiritani, Teruo Kirikae, Keiji Funatogawa

**Affiliations:** 1 Tochigi Prefectural Institute of Public Health and Environmental Science, Utsunomiya, Tochigi Japan; 2 Department of Infectious Diseases, Research Institute, National Center for Global Health and Medicine, Tokyo, Japan; 3 Pathogenic Microbe Laboratory, Research Institute, National Center for Global Health and Medicine, Tokyo, Japan; 4 Present Address: Department of Microbiology, Juntendo University School of Medicine, Tokyo, Japan

**Keywords:** Foreign-born, Lineage, Whole genome sequencing, CASTB, Japan

## Abstract

**Background:**

Foreign-born patients with tuberculosis (TB) may introduce globally disseminated isolates of *Mycobacterium tuberculosis* into large cities in Japan. The risk of dissemination of these isolates into local regions, however, has not been determined. This study analyzed the molecular epidemiology of *M. tuberculosis* isolates obtained from TB patients living in a local region of Japan.

**Methods:**

Whole genome sequences of 169 *M. tuberculosis* isolates, obtained from 148 Japanese-born and 21 foreign-born patients living in Tochigi, Japan, were analyzed using the **C**omprehensive **a**nalysis **s**erver for the *Mycobacterium* **t** *u* ***b*** *erculosis* complex (CASTB).

**Results:**

The 169 isolates were clustered into four clades; Lineage 2 (111 isolates 65.7%), Lineage 4 (43 isolates, 25.4%), Lineage 1 (13 isolates, 7.7%), and Lineage 3 (2 isolates, 1.2%). Of the 111 isolates belonging to Lineage 2, 79 (71.2%) were of the atypical Beijing sub-genotype. Of the 13 Lineage 1 isolates, nine (69.2%) were from foreign-born patients. The isolates belonging to Lineage 4 were further clustered into three clades, two containing isolates shared by both Japanese- and foreign-born patients. The two isolates belonging to Lineage 3 were obtained from foreign-born patients.

**Conclusions:**

The genotypic diversity of *M. tuberculosis* in a local region of Japan is increased primarily by the presence of isolates obtained from foreign-born patients.

**Electronic supplementary material:**

The online version of this article (doi:10.1186/s12879-017-2457-y) contains supplementary material, which is available to authorized users.

## Background

The World Health Organization has estimated that, in 2014, 9.6 million people worldwide were newly infected with tuberculosis (TB) and that 1.5 million people died of this disease [1]. Most Asian countries, except for Japan, the Republic of Korea and Singapore, have been categorized as countries with a high or relatively high TB burden, defined as ≥100 per 100,000 population [1]. The prevalence of TB in large Japanese cities, such as Tokyo, is more than twice that of rural areas [2], with the higher prevalence likely due to the greater presence of foreign-born TB patients in large cities. These foreign-born individuals may carry strains of *M. tuberculosis* differing from those present in Japan, reflecting the global diversity of this bacterial species [3]. These patients may therefore introduce globally disseminated isolates of *M. tuberculosis* into Japan, especially strains from countries with high TB burdens [3]. Less is known, however, about risk factors for TB in local regions throughout Japan.

Recent molecular epidemiologic studies of *M. tuberculosis* have provided important information on epidemic and drug-resistant strains and on their relationships [4, 5]. *M. tuberculosis* strains have evolved and become distributed globally during human migrations in response to historical events including the industrial revolution and the two World Wars [5]. Epidemiological analytical methods used to assess TB strains have included determination of variable numbers of tandem repeats, spoligotyping, IS6110-based restriction fragment length polymorphism (RFLP) typing, lineage analysis based on long sequence polymorphisms (LSP), and Beijing typing [6–10]. In addition, phylogenetic analysis based on single nucleotide polymorphism (SNP) concatemers is becoming a powerful tool for higher resolution analysis in the era of next-generation sequencing [11–13]. Detection of mutations associated with drug resistance is also important due to the increasing number of patients with drug-resistant TB [14].

The comprehensive analysis server for the *Mycobacterium* *t* *u* *b* *erculosis* complex (CASTB) is a recently developed web server (http://castb.ri.ncgm.go.jp/CASTB/) [15] that can analyze whole-genome sequencing (WGS) data generated by next-generation sequencers (NGS). Analysis using this server can automatically generate conventional epidemiological results, predict drug resistance, and determine phylogeny based on SNP concatemers. Phylogenetic analysis by CASTB based on SNP concatemers allows high-resolution analysis of relationships among *M. tuberculosis* isolates. CASTB can determine genetic lineages from WGS data, especially from virtual LSP typing. LSP analysis has been used to classify *M. tuberculosis* complexes into six major lineages. Moreover, CASTB can distinguish between isolates belonging to “typical” and “atypical” Beijing sub-lineages, as well as detecting mutations associated with drug resistance and predicting the drug susceptibility of strains.

Tochigi Prefecture of Japan is located 100 km north of Tokyo, in the center of Honshu, the main island of Japan. This prefecture, which consists of three regions, classified as the northern, central and southern regions, has an area of 6,400 km^2^ and a population of 2 million people (Additional file 1: Figure S1). To assess the molecular epidemiology of *M. tuberculosis* in a localized area, this study used CASTB to analyze whole genome sequences of *M. tuberculosis* clinical isolates in Tochigi prefecture.

## Methods

### Patients and clinical isolates

A total of 169 clinical isolates of *M. tuberculosis* were obtained from 169 TB patients residing in Tochigi Prefecture. In Japan, the Law Concerning the Prevention of Infections and Medical Care for Patients of Infections mandates that local public health institutions, such as the Tochigi Prefectural Institute of Public Health and Environmental Science (Tochigi IPH), collect all *M. tuberculosis* isolates obtained from TB patients, as well as anonymized clinical information about these patients. The Tochigi IPH began to collect *M. tuberculosis* clinical isolates in 2007. Isolates obtained in 2007 and in 2013 were therefore analyzed in this study. Of the 169 patients, 31, including one foreign-born patient, were living in the northern part of Tochigi; 83, including 13 foreign-born patients, were living in the central part; and 55, including seven foreign-born patients, were living in the southern part (Additional file 2: Table S1). Of these isolates, 87 and 82 were isolated in 2007 and 2013, respectively (Table 1). Of the 87 isolates obtained in 2007, 11 (12.6%) were from foreign-born patients (Table 1) and 60 (69.0%) were from males (Additional file 3: Table S2). Of the 82 isolates obtained in 2013, 10 (12.2%) were from foreign-born patients (Table 1) and 44 (53.7%) were from males (Additional file 3: Table S2). The average age of the 169 patients was 62.7 years (range, 1.7–98.5 years), 37.9 years for patients with *M. tuberculosis* Lineage 1; 69.4 and 57.1 years for patients with Ancestral and Modern Lineage 2, respectively; 35.1 years for patients with Lineage 3; and 63.3 years for patients with Lineage 4 (Fig. 4). The 21 foreign-born patients (Table 1, parentheses) were from nine countries/regions, including four each from Taiwan and the Philippines; three from Korea; two each from China, Nepal, Peru, and Thailand; and one each from Brazil and Vietnam. Information about patients and isolates, including dates and countries/regions of birth, gender, areas of residence, and results of drug susceptibility testing, was obtained from the National Epidemiological Surveillance of Infectious Diseases (NESID), an active infectious disease surveillance system in Japan that collects reports about patients with various infectious diseases [16].

**Table 1:** Lineage distribution of *M. tuberculosis* isolates in 2007 and 2013

	Lineage/Beijing typing					
	Lineage 1	Lineage 2		Lineage 3	Lineage 4	Total
		Ancestral (Atypical)	Modern (Typical)			
Total	13 (9)	111 (4)		2 (2)	43 (6)	169 (21)
		79 (3)	32 (1)			
2007	8 (5) [9.2%]	44 (3) [50.6%]	13 (0) [14.9%]	0 (0) [0.0%]	22 (3) [25.3%]	87 (11) [100.0%]
2013	5 (4) [6.1%]	35 (0) [42.7%]	19 (1) [23.2%]	2 (2) [2.4%]	21 (3) [25.6%]	82 (10) [100.0%]

Numbers in parentheses represent numbers of foreign-born TB patients

Numbers in brackets represent percentages of foreign-born TB patients

### *DNA preparation and whole genome sequencing of* M. tuberculosis *isolates*

Genomic DNA was extracted from all isolates by standard procedures [14]. Paired-end multiplexed Illumina sequencing was performed using MiSeq (Illumina Inc., San Diego, CA, USA), followed by mapping of sequence reads [17].

### CASTB analysis

WGS data, generated by NGS, were analyzed using the CASTB web server [15]. Conventional epidemiological results, predicted resistance against drugs (ciprofloxacin, ethambutol, pyrazinamide, rifampin and streptomycin), and lineage analysis based on virtual LSP and SNP concatemers, as well as spoligotyping, were automatically obtained from sequence data.

### Phylogenetic analyses using maximum likelihood (ML) and neighbor-joining (NJ) methods

The ML and NJ methods were used for phylogenetic analyses. The identified SNPs were concatenated to obtain concatenated SNP sequences for alignment with MAFFT [18]. After obtaining the best model for the ML tree, a phylogenetic tree was generated by aligning SNPs using a general time reversible model, with MEGA version 7, PhyML3.0 [19] and FigTree v1.4.0. software (http://tree.bio.ed.ac.uk/software/figtree/).

### Statistical analyses

Data were summarized as mean, median, and/or range, as appropriate, and compared using Fisher’s exact test or the nonparametric Student’s *t* test. All tests were two-sided, with *p* < 0.05 considered statistically significant.

## Results

### Phylogenetic and lineage analyses

**Fig. 1:**
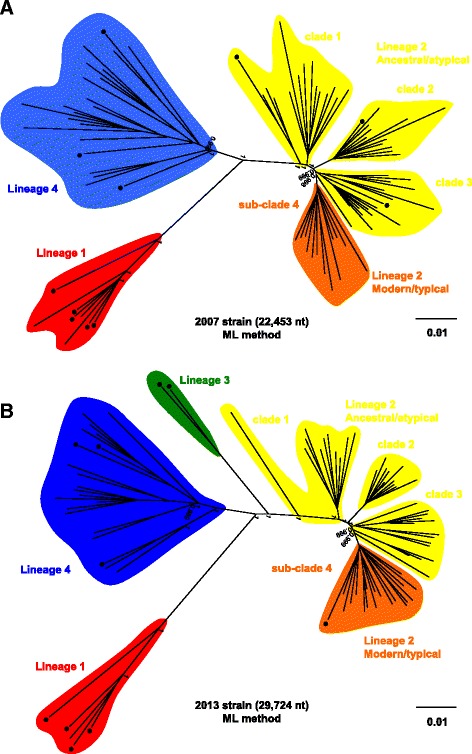
ML phylogenetic trees based on SNP concatenated sequence alignments of clinical isolates. ML phylogenetic trees of 169 clinical isolates based on whole genome analysis. Radial rectangular tree layouts are shown for the years of *M. tuberculosis* isolation, **a** 2007 and **b** 2013. Bootstrap values are shown in the phylogenetic trees. Clades show Lineage 1 (*red*), Lineage 2 (*orange* [Modern/typical] and *yellow* [Ancestral/atypical]), Lineage 3 (*green*) and Lineage 4 (*blue*). Scale bars indicate nucleotide substitutions per site. *Black circles* indicate foreign-born TB patients

**Fig. 2:**
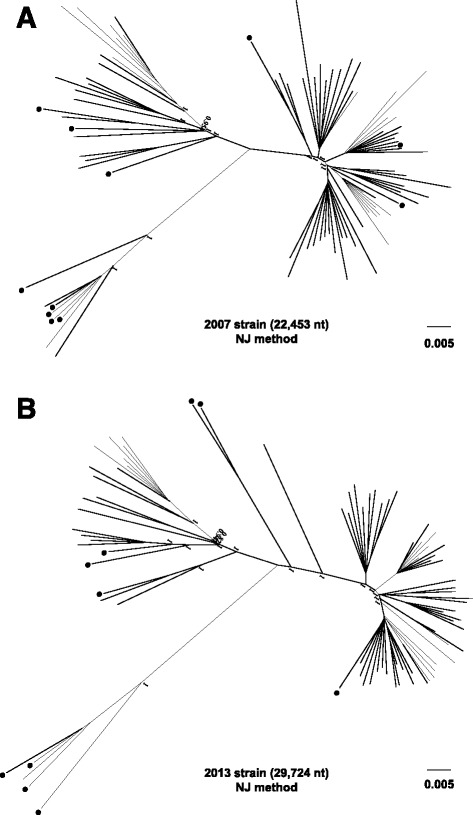
NJ phylogenetic trees based on SNP concatenated sequence alignments of clinical isolates. NJ phylogenetic trees of the 169 clinical isolates based on whole genome analysis. Radial rectangular tree layouts are shown for the years of *M. tuberculosis* isolation, **a** 2007 and **b** 2013. Bootstrap values are shown in the phylogenetic trees. Scale bars indicate nucleotide substitutions per site. *Black circles* indicate foreign-born TB patients

ML trees were constructed from the 169 *M. tuberculosis* isolates obtained from patients registered in the NESID in 2007 and 2013 (Fig. 1a and b). These ML trees were congruent with the trees constructed by the neighbor-joining method for each of these years (Fig. 2a and b). Phylogenetic analysis revealed four clades in good agreement with four of the lineages (Lineages 1–4) categorized by genotyping based on LSP (Figs. 1 and 2). Of the 169 isolates, 111 (65.7%) belonged to Lineage 2 (East Asian or Beijing), 43 (25.4%) to Lineage 4 (Euro-American), 13 (7.7%) to Lineage 1 (Indo-Oceanic), and two (1.2%) to Lineage 3 (East African-Indian) (Table 1). Of the 111 isolates belonging to Lineage 2, 32 (28.8%) belonged to the typical (modern) Beijing sub-genotype, and 79 (71.2%) to the atypical (ancestral) Beijing sub-genotype. Fifty-seven (65.5%) of the 87 isolates obtained in 2007, and 54 (65.9%) of the 82 isolated in 2013 belonged to Lineage 2, with 44 (50.6%) and 35 (42.7%), respectively, belonging to the atypical Beijing sub-genotype (Table 1). The percentage of isolates belonging to the typical Beijing sub-genotype was higher in 2013 (19/82, 23.2%) than in 2007 (13/87, 14.9%).

**Fig. 3:**
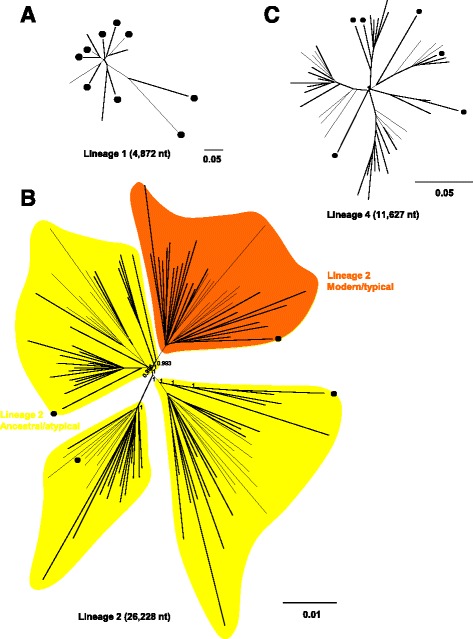
ML phylogenetic trees based on SNP concatenated sequence alignments of clinical isolates. ML phylogenetic trees of the 169 clinical isolates based on whole genome analysis. Radial rectangular tree layouts are shown for Lineages 1 (**a**), 2 (**b**) and 4 (**c**). Bootstrap values are shown in the phylogenetic trees. Clades show Lineage 2 Modern/typical (*orange*) and Ancestral/atypical (*yellow*). Scale bars indicate nucleotide substitutions per site. *Black circles* indicate foreign-born TB patients

Three ML trees were constructed from isolates belonging to Lineages 1, 2 and 4, respectively (Fig. 3). The 13 isolates belonging to Lineage 1 did not form any sub-clades (Fig. 3a). The 111 isolates belonging to Lineage 2 could be divided into four sub-clades, containing 26, 22, 31 and 32 isolates, respectively (Fig. 3b). Isolates belonging to clades 1, 2 and 3 were of the atypical (ancestral) Beijing sub-genotype, with the isolates belonging to sub-clade 4 being of the typical Beijing sub-genotype (Fig. 3b). Forty-three isolates belonged to Lineage 4 (Fig. 3c). Both isolates belonging to Lineage 3 were detected in 2013 (Fig.1a), with none detected in 2007 (Fig. 1b).

### Lineage comparison

#### Isolates from foreign- and Japanese-born patients

Of the 169 isolates, 21 (12.4%) were from foreign-born patients, with nine, four, two, and six belonging to Lineages 1, 2, Lineage 3, and 4, respectively (Table 1). Of the 13 Lineage 1 isolates nine (69.2%) were from foreign-born patients (Table 1 and Fig. 3a), including three patients from the Philippines, two each from Taiwan and Thailand, and one each from Nepal and Vietnam. Of the 111 isolates belonging to Lineage 2, 107 (96.4%) were from Japan-born patients and only four (3.6%) from foreign-born patients (Table 1), with one each of the latter belonging to each of the four sub-clades of Lineage 2 (Fig. 3b). One isolate, belonging to the typical sub-genotype of Lineage 2, was obtained from a patient born in China, whereas the other three were from patients born in Korea, Taiwan, and China, respectively. The two Lineage 3 isolates detected in 2013 (Table 1) were obtained from foreign-born patients, a man in his twenties from Nepal and a woman in her forties from the Philippines, both of whom were living in the southern region of Tochigi. Of the 43 isolates belonging to Lineage 4, six (14.0%) were from foreign-born patients, including two each from Korea and Peru and one each from Brazil and Thailand (Fig. 3c).

#### Elderly and nonelderly patients

**Fig. 4:**
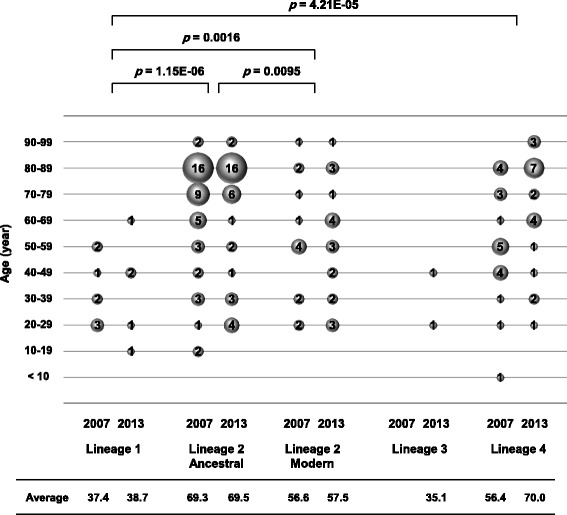
Associations between age distribution of patients and *Mycobacterium tuberculosis* lineages. The *balloon plot* indicates patient age at isolation of *M. tuberculosis* strains, with the size of the balloons representing the number of patients. The average ages appear below the graph

Figure 4 shows the age distribution from which isolates of each lineage were obtained. Among patients infected with Lineage 2 isolates, those infected with Ancestral strains were significantly older than those infected with Modern strains (*p* = 0.0095). Of the 79 patients with Lineage 2-Ancestral isolates and the 22 with Lineage 4 isolates in 2007 (Table 1), 78 and 20, respectively developed TB at the indicated ages (Fig. 4). Japan-born patients with Lineage 1 isolates were significantly younger than patients with Lineage 2-Ancestral (*p* = 1.15E-06), Lineage 2-Modern (*p* = 0.0016), and Lineage 4 (*p* = 4.21E-05) isolates. Patients with isolates belonging to Lineage 1, both Japan- and foreign-born, were also significantly younger than either Japan-born patients with Lineage 2-Ancestral (*p* = 9.38E-04) or Lineage 4 (*p* = 1.56E-03) isolates. Both patients with Lineage 3 isolates were relatively young, one in his twenties and the other in her forties.

#### Residential regions of patients (northern, central and southern parts of Tochigi prefecture)

Both patients infected with Lineage 3 isolates lived in the southern part of Tochigi prefecture. There were no other relationships between the Lineages of isolates and residential regions (Additional file 4: Figure S2 and Additional file 2: Table S1).

#### Patient gender

There were twice as many male as female patients in 2007, with the numbers of males infected with Lineage 1, 2, and 4 isolates in that year being higher than those of females (Additional file 3: Table S2). The number of male patients was slightly higher than the number of females in 2013 (Additional file 3: Table S2).

### Drug susceptibility

CASTB analysis showed that, of the 169 isolates, 11 (6.5%) were resistant to at least one drug, including five resistant to isoniazid, three resistant to streptomycin, and one each resistant to ciprofloxacin, isoniazid + streptomycin, and isoniazid + streptomycin + ciprofloxacin. Of the five isoniazid-resistant isolates, three belonged to Lineage 1 and two to Lineage 4. The three isolates belonging to Lineage 1 were from three patients born in the Philippines, whereas the two belonging to Lineage 4 were from two Japan-born patients. One isolate resistant to both isoniazid and streptomycin belonged to Lineage 2 and was from a patient born in China. The isolate resistant to ciprofloxacin belonged to Lineage 4 and was from a Japan-born patient. One of the isolates resistant to isoniazid, streptomycin and ciprofloxacin belonged to Lineage 2 and was from a Japan-born patient. Collectively, of the 11 drug-resistant isolates, four were from foreign-born patients, with the percentage of drug-resistant isolates being significantly higher in foreign-born than in Japan-born patients (*p* = 0.033).

NESID reports included the results of drug susceptibility tests for 86 (50.9%) of 169 isolates, not for the other 83. Of the 86 isolates, three were resistant to at least one drug, with one each resistant to isoniazid, isoniazid + ethionamide, and isoniazid + streptomycin + levofloxacin. The drug susceptibility profiles of these isolates essentially agreed with those of CASTB analysis (Additional file 5: Table S3).

## Discussion

This study clearly showed that foreign-born TB patients affect the genetic diversity of *M. tuberculosis* isolates in Tochigi Prefecture, a local region of Japan. This conclusion was supported by results showing that most of the Lineage 1 isolates were from foreign-born patients, as were both of the Lineage 3 isolates. We previously reported that foreign-born TB patients also affect the genetic diversity of isolates in the center of metropolitan Tokyo [3]. These results indicate that foreign-born TB patients have an impact on the nationwide genetic diversity of *M. tuberculosis* in Japan.

TB epidemiology has been analyzed in foreign-born patients living in countries with low TB prevalence, including Madrid and Catalonia, Spain [20], the USA (http://www.ingentaconnect.com/), Canada [21], Switzerland [22], and Germany [23]. Although most of these studies found little transmission of *M. tuberculosis* from foreign-born patients to native-born residents, our phylogenetic and lineage analyses predicted *M. tuberculosis* transmission among foreign-born and Japan-born TB patients. The difference in the impact of foreign-born TB patients on TB epidemiology may be due to the prevalence of TB being higher in Japan, a country with a middle TB-burden, than in developed countries, with a low TB-burden, as well as to differences in employment and lifestyles.

These findings also showed the globalization of *M. tuberculosis* transmission in a local region of Japan. TB isolates from foreign-born patients may increase TB genetic diversity in Tochigi Prefecture. Although no direct evidence has shown that these isolates from foreign-born patients in Tochigi are a risk factor for TB transmission, similar findings were reported for TB isolates from foreign-born and Japan-born TB patients in Tokyo [3]. Of the 13 isolates belonging to Lineage 1, nine were from patients born in other Asian countries, including seven from Southeast Asian countries/regions, where Lineage 1 is predominant [6, 24]. In addition, three of the 43 Lineage 4 isolates were from patients born in South America, where Lineage 4 is predominant [6, 24]. Of individuals living in Tochigi in 2007 and 2013, only 1.7% and 1.5%, respectively, were registered foreigners (http://www.pref.tochigi.lg.jp/f04/documents/h27jumin-sityoubetu.pdf; the Tochigi prefectural websites in Japanese), whereas 12.6% and 12.2%, respectively, of isolates obtained nationwide during these 2 years were from foreign-born patients. Beginning in 2012, more than 1,000 foreign-born TB patients are newly registered annually in Japan, with the numbers continuing to increase (http://www.mhlw.go.jp/bunya/kenkou/kekkaku-kansenshou03/14.html; in Japanese). The globalization and diversity of *M. tuberculosis* are increasing in Japan, making molecular epidemiology studies of TB isolates obtained from both foreign-born and native-born patients necessary.

## Conclusion

In conclusion, whole genome analysis of 169 *M. tuberculosis* isolates in Tochigi prefecture using CASTB revealed the genetic diversity of *M. tuberculosis* isolates in a local area of Japan, as well as the impact of foreign-born TB patients. Isolates from foreign-born TB patients were apparently not transmitted extensively to Japan-born residents, these foreign-born patients carry a broader lineage of TB strains. CASTB is a useful tool for assessing the epidemiology of *M. tuberculosis*.

## Additional files

Additional file 1: Figure S1. Geographical location of Tochigi Prefecture in Japan. Tochigi is one of the inland prefectures of the Northern portion of the Kanto region. Its population on March 1, 2007, was 2,014,931 persons. The area of Tochigi prefecture is approximately 6,400 km^2^, making it the 20th largest in Japan, but the largest in the Kanto region. (PPTX 127 kb)

Additional file 2: Table S1. Geographic analysis of lineages of *M. tuberculosis* isolates obtained from foreign- and Japanese-born patients living in the North, Central, and South regions of Tochigi Prefecture. (DOCX 31 kb)

Additional file 3: Table S2. Associations between patient gender and lineages of *M. tuberculosis* isolates obtained from foreign- and Japanese-born patients. (DOCX 31 kb)

Additional file 4: Figure S2. Associations between patient gender and *Mycobacterium tuberculosis* lineages. The stacked bar charts show the percentages of *M. tuberculosis* lineages by (A) countries of birth and (B) sampling period. The numbers of isolates are indicated on the graphs. (PPTX 133 kb)

Additional file 5: Table S3. Drug susceptibility and resistance of *M. tuberculosis* isolates. (DOCX 31 kb)

## Abbreviations

CASTB: The comprehensive analysis server for the *Mycobacterium tuberculosis* complex
LSP: Lineage analysis based on long sequence polymorphisms
ML: Maximum likelihood
NESID: National epidemiological surveillance of infectious diseases
NGS: Next-generation sequencers
NJ: Neighbor-joining
SNP: Single nucleotide polymorphism
TB: Tuberculosis
WGS: Whole-genome sequencing
