# Supplementary material for: Genetic diversity of Mycobacterium tuberculosis isolates from Tochigi prefecture, a local region of Japan
Source: BMC Infect Dis. 2017 May 25;17:365. doi: 10.1186/s12879-017-2457-y (PMC5445273; doi:10.1186/s12879-017-2457-y)
Supplement: Supplementary file 1 — Geographical location of Tochigi Prefecture in Japan. Tochigi is one of the inland prefectures of the Northern portion of the Kanto region. Its population on March 1, 2007, was 2,014,931 persons. The area of Tochigi prefecture is approximately 6,400 km2, making it the 20th largest in Japan, but the largest in the Kanto region. (PPTX 127 kb) [file 12879_2017_2457_MOESM1_ESM.pptx]

## Slide 1
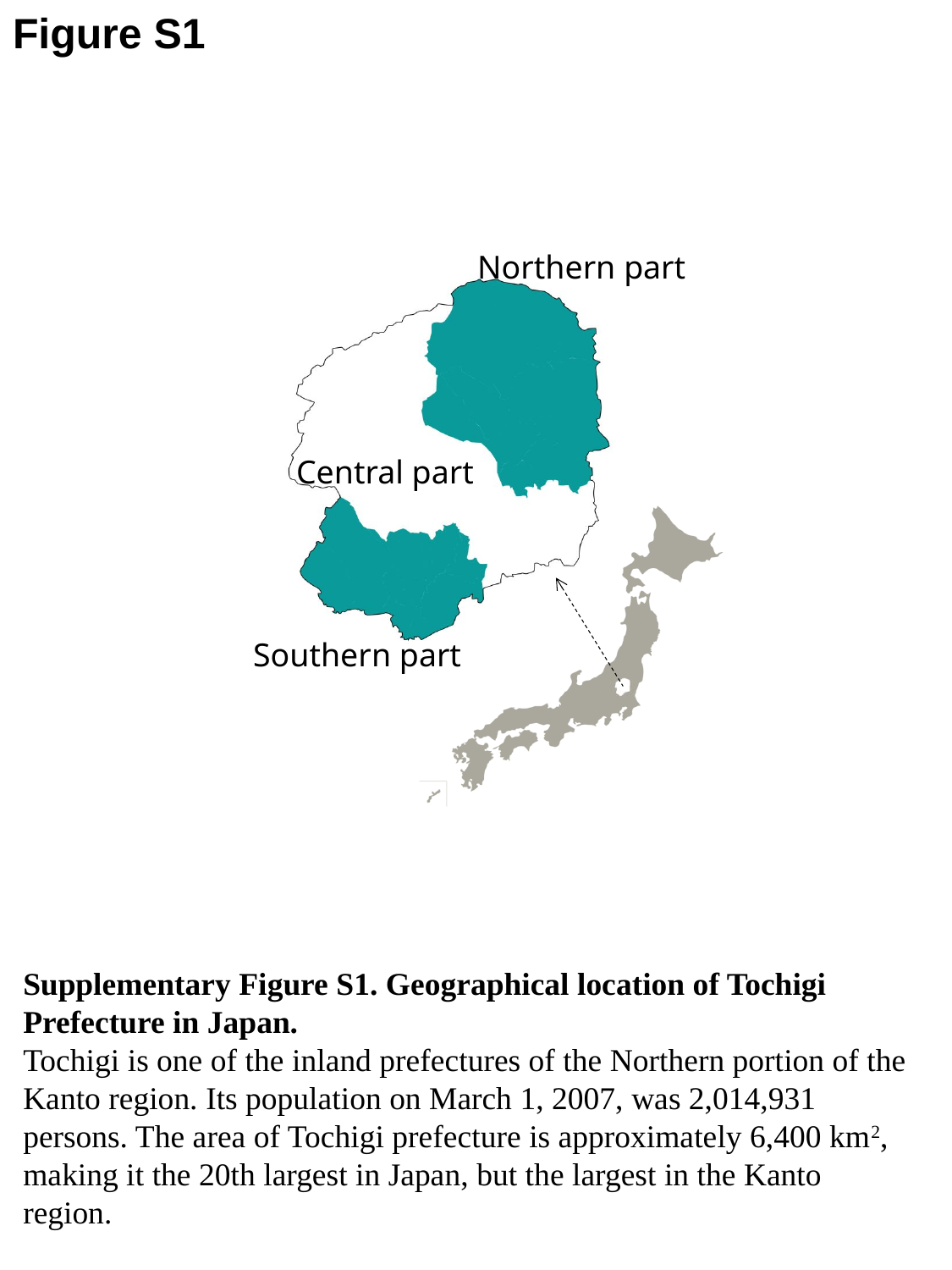

Figure S1
Northern part
 Central part
Southern part
Supplementary Figure S1. Geographical location of Tochigi Prefecture in Japan.
Tochigi is one of the inland prefectures of the Northern portion of the Kanto region. Its population on March 1, 2007, was 2,014,931 persons. The area of Tochigi prefecture is approximately 6,400 km2, making it the 20th largest in Japan, but the largest in the Kanto region.
