# Supplementary material for: Genetic diversity of Mycobacterium tuberculosis isolates from Tochigi prefecture, a local region of Japan
Source: BMC Infect Dis. 2017 May 25;17:365. doi: 10.1186/s12879-017-2457-y (PMC5445273; doi:10.1186/s12879-017-2457-y)
Supplement: Supplementary file 2 — Geographic analysis of lineages of M. tuberculosis isolates obtained from foreign- and Japanese-born patients living in the North, Central, and South regions of Tochigi Prefecture. (DOCX 31 kb) [file 12879_2017_2457_MOESM2_ESM.docx]

Table S1. Geographic analysis of lineages of *M. tuberculosis* isolates obtained from foreign- and Japanese-born patients living in the North, Central, and South regions of Tochigi Prefecture.

|  | | Lineage/Beijing typing | | | | | |
| --- | --- | --- | --- | --- | --- | --- | --- |
|  |  | 1 | 2 | | 3 | 4 | Total |
|  |  |  | Ancestral  (Atypical) | Modern  (Typical) |  |  |  |
| 2007 | North | 2 (1) | 7 | 2 | 0 | 3 | 14 (1) |
|  | Central | 5 (4) | 24 (2) | 3 | 0 | 8 (2) | 40 (8) |
|  | South | 1 | 13 (1) | 8 | 0 | 11 (1) | 33 (2) |
| 2013 | North | 0 | 6 | 5 | 0 | 6 | 17 |
|  | Central | 3 (2) | 21 | 9 (1) | 0 | 10 (2) | 43 (5) |
|  | South | 2 (2) | 8 | 5 | 2 (2) | 5 (1) | 22 (5) |

*Numbers in parentheses represent the numbers of foreign-born TB patients.
