# Supplementary material for: Genetic diversity of Mycobacterium tuberculosis isolates from Tochigi prefecture, a local region of Japan
Source: BMC Infect Dis. 2017 May 25;17:365. doi: 10.1186/s12879-017-2457-y (PMC5445273; doi:10.1186/s12879-017-2457-y)
Supplement: Supplementary file 3 — Associations between patient gender and lineages of M. tuberculosis isolates obtained from foreign- and Japanese-born patients. (DOCX 31 kb) [file 12879_2017_2457_MOESM3_ESM.docx]

Table S2. Associations between patient gender and lineages of *M. tuberculosis* isolates obtained from foreign- and Japanese-born patients.

|  | | Lineage/Beijing typing | | | | | |
| --- | --- | --- | --- | --- | --- | --- | --- |
|  |  | 1 | 2 | | 3 | 4 | Total |
|  |  |  | Ancestral  (atypical) | Modern  (typical) |  |  |  |
| 2007 | Male | 6 (3) | 29 | 9 | 0 | 16 (2) | 60 (5) |
|  | Female | 2 (2) | 15 (3) | 4 | 0 | 6 (1) | 27 (6) |
| 2013 | Male | 1 (1) | 20 | 11 | 1 (1) | 11 (1) | 44 (3) |
|  | Female | 4 (3) | 15 | 8 (1) | 1 (1) | 10 (2) | 38 (7) |

*Numbers in parentheses represent the numbers of foreign-born patients.
