# Supplementary material for: Genetic diversity of Mycobacterium tuberculosis isolates from Tochigi prefecture, a local region of Japan
Source: BMC Infect Dis. 2017 May 25;17:365. doi: 10.1186/s12879-017-2457-y (PMC5445273; doi:10.1186/s12879-017-2457-y)
Supplement: Supplementary file 4 — Associations between patient gender and Mycobacterium tuberculosis lineages. The stacked bar charts show the percentages of M. tuberculosis lineages by (A) countries of birth and (B) sampling period. The numbers of isolates are indicated on the graphs. (PPTX 133 kb) [file 12879_2017_2457_MOESM4_ESM.pptx]

## Slide 1
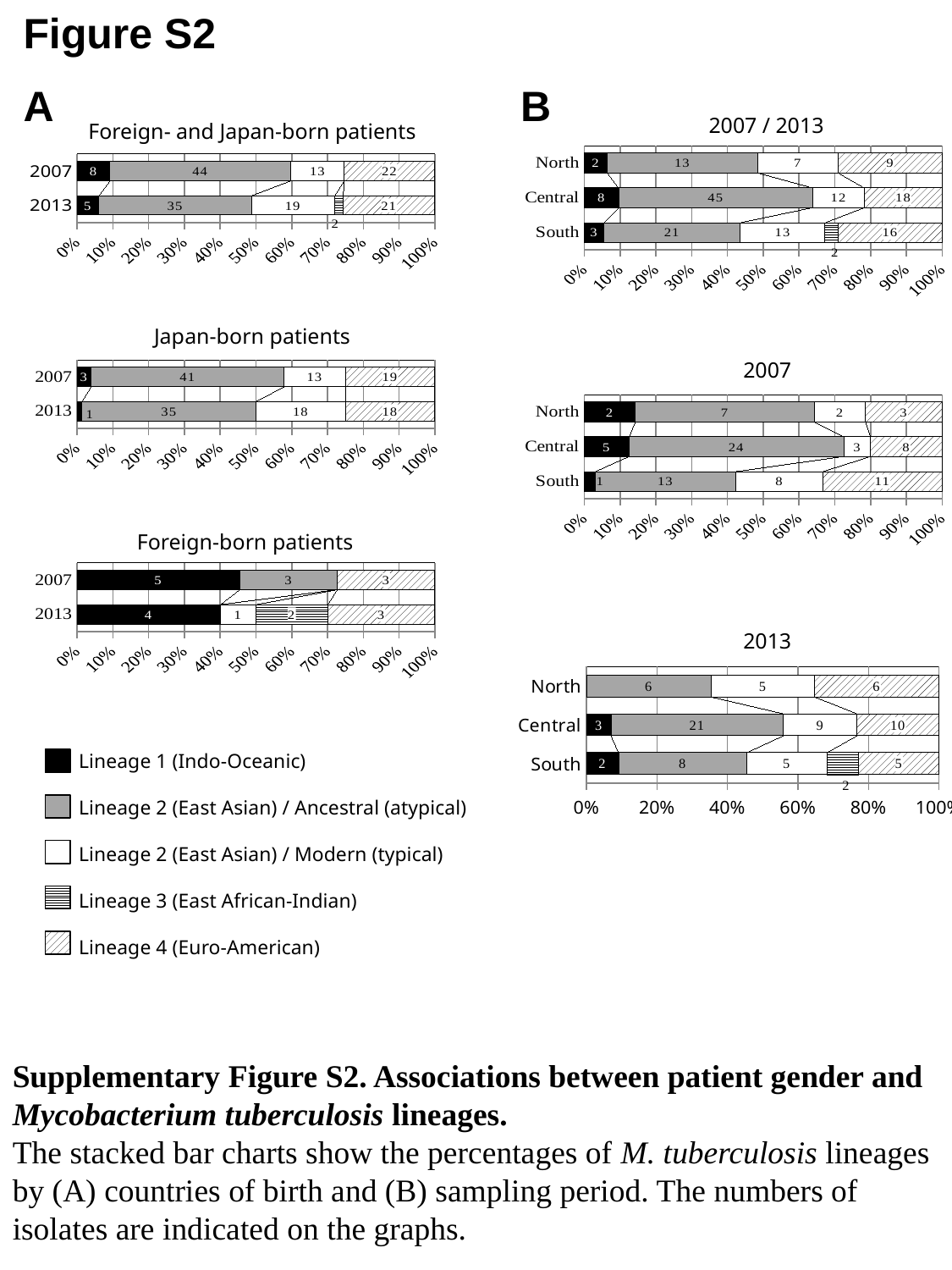

Figure S2
A
B
2007 / 2013
Foreign- and Japan-born patients
### Chart
| Category | Lineage 1
Indo-Oceanic | Lineage 2
East-Asian/Ancestral
 | Lineage 2
East-Asian/Modern | Lineage 3
East-African-Indian | Lineage 4
Euro-American |
|---|---|---|---|---|---|
| South | 3.0 | 21.0 | 13.0 | 2.0 | 16.0 |
| Central | 8.0 | 45.0 | 12.0 | None | 18.0 |
| North | 2.0 | 13.0 | 7.0 | None | 9.0 |
### Chart
| Category | Lineage 1
Indo-Oceanic | Lineage 2
East-Asian/Ancestral
 | Lineage 2
East-Asian/Modern | Lineage 3
East-African-Indian | Lineage 4
Euro-American |
|---|---|---|---|---|---|
| 2013.0 | 5.0 | 35.0 | 19.0 | 2.0 | 21.0 |
| 2007.0 | 8.0 | 44.0 | 13.0 | 0.0 | 22.0 |Japan-born patients
2007
### Chart
| Category | Lineage 1
Indo-Oceanic | Lineage 2
East-Asian/Ancestral
 | Lineage 2
East-Asian/Modern | Lineage 3
East-African-Indian | Lineage 4
Euro-American |
|---|---|---|---|---|---|
| 2013.0 | 1.0 | 35.0 | 18.0 | 0.0 | 18.0 |
| 2007.0 | 3.0 | 41.0 | 13.0 | 0.0 | 19.0 |
### Chart
| Category | Lineage 1
Indo-Oceanic | Lineage 2
East-Asian/Ancestral
 | Lineage 2
East-Asian/Modern | Lineage 3
East-African-Indian | Lineage 4
Euro-American |
|---|---|---|---|---|---|
| South | 1.0 | 13.0 | 8.0 | None | 11.0 |
| Central | 5.0 | 24.0 | 3.0 | None | 8.0 |
| North | 2.0 | 7.0 | 2.0 | None | 3.0 |Foreign-born patients
### Chart
| Category | Lineage 1
Indo-Oceanic | Lineage 2
East-Asian/Ancestral
 | Lineage 2
East-Asian/Modern | Lineage 3
East-African-Indian | Lineage 4
Euro-American |
|---|---|---|---|---|---|
| 2013.0 | 4.0 | 0.0 | 1.0 | 2.0 | 3.0 |
| 2007.0 | 5.0 | 3.0 | 0.0 | 0.0 | 3.0 |2013
### Chart
| Category | Lineage 1
Indo-Oceanic | Lineage 2
East-Asian/Ancestral
 | Lineage 2
East-Asian/Modern | Lineage 3
East-African-Indian | Lineage 4
Euro-American |
|---|---|---|---|---|---|
| South | 2.0 | 8.0 | 5.0 | 2.0 | 5.0 |
| Central | 3.0 | 21.0 | 9.0 | None | 10.0 |
| North | None | 6.0 | 5.0 | None | 6.0 |Lineage 1 (Indo-Oceanic)
Lineage 2 (East Asian) / Ancestral (atypical)
Lineage 2 (East Asian) / Modern (typical)
Lineage 3 (East African-Indian)
Lineage 4 (Euro-American)
Supplementary Figure S2. Associations between patient gender and Mycobacterium tuberculosis lineages.
The stacked bar charts show the percentages of M. tuberculosis lineages by (A) countries of birth and (B) sampling period. The numbers of isolates are indicated on the graphs.
