# Supplementary material for: Genetic diversity of Mycobacterium tuberculosis isolates from Tochigi prefecture, a local region of Japan
Source: BMC Infect Dis. 2017 May 25;17:365. doi: 10.1186/s12879-017-2457-y (PMC5445273; doi:10.1186/s12879-017-2457-y)
Supplement: Supplementary file 5 — Drug susceptibility and resistance of M. tuberculosis isolates. (DOCX 31 kb) [file 12879_2017_2457_MOESM5_ESM.docx]

Table S3. Drug susceptibility and resistance.

| Isolate ID# | CASTB | | | | NESID | | | |
| --- | --- | --- | --- | --- | --- | --- | --- | --- |
|  | INH | SM | EB | Others | INH | SM | EB | Others |
| 13-048 | R | R |  | CFX | R | R |  | LVFT |
| 13-021 | R |  |  |  | R |  |  | TH |
| 08-010 | R |  |  |  | R |  |  |  |

Abbreviations: INH, isoniazid; SM, streptomycin; EB, ethambutol; CFX, cefoxitin; LVFT, levofloxacin; TH, ethionamide
